# Induction of the Viable but Nonculturable State of *Ralstonia solanacearum* by Low Temperature in the Soil Microcosm and Its Resuscitation by Catalase

**DOI:** 10.1371/journal.pone.0109792

**Published:** 2014-10-08

**Authors:** Hyun Gi Kong, Ju Young Bae, Hyoung Ju Lee, Hae Jin Joo, Eun Joo Jung, Eunsook Chung, Seon-Woo Lee

**Affiliations:** Department of Applied Bioscience, Dong-A University, Busan, Republic of Korea; University of Wisconsin-Milwaukee, United States of America

## Abstract

*Ralstonia solanacearum* is the causal agent of bacterial wilt on a wide variety of plants, and enters a viable but nonculturable (VBNC) state under stress conditions in soil and water. Here, we adopted an artificial soil microcosm (ASM) to investigate the VBNC state of *R. solanacearum* induced by low temperature. The culturability of *R. solanacearum* strains SL341 and GMI1000 rapidly decreased at 4°C in modified ASM (mASM), while it was stably maintained at 25°C in mASM. We hypothesized that bacterial cells at 4°C in mASM are viable but nonculturable. Total protein profiles of SL341 cells at 4°C in mASM did not differ from those of SL341 culturable cells at 25°C in mASM. Moreover, the VBNC cells maintained in the mASM retained respiration activity. Catalase treatment effectively restored the culturability of nonculturable cells in mASM, while temperature increase or other treatments used for resuscitation of other bacteria were not effective. The resuscitated *R. solanacearum* from VBNC state displayed normal level of bacterial virulence on tomato plants compared with its original culturable bacteria. Expression of *omp*, *oxyR*, *rpoS*, *dps*, and the 16S rRNA gene quantified by RT-qPCR did not differ significantly between the culturable and VBNC states of *R. solanacearum*. Our results suggested that the VBNC bacterial cells in mASM induced by low temperature exist in a physiologically unique state.

## Introduction

*Ralstonia solanacearum* is a plant pathogenic bacterium that causes severe bacterial wilt on over 400 plant species across 50 families. Bacterial wilt by *R. solanacearum* causes significant yield losses of many economically important crops [1]. However, effective management practices are not available for bacterial wilt on a variety of plant species. Additionally, chemical control cannot be used to eliminate the bacterial pathogen from soils and waters. Cultivation of resistant cultivars is an option, but resistant stocks are available for only specific plant species [2]. This bacterium can survive in the soil or water for long periods of time and infects host plants through the roots [3], [4], [5]. It has been documented that *R. solanacearum* survives in water or soil for extended periods of time, and can cause disease outbreaks when environmental conditions turn favorable for infection [5], [6]. Therefore, long-term survival of *R. solanacearum* could be a threat to crop cultivation, which complicates bacterial wilt management.

Investigation of the long-term survival of *R. solanacearum* is dependent on bacterial cultivation. However, cultivation-dependent bacterial detection has some limitations. For example, Gram-negative bacteria exposed to stresses cannot be cultivated, but stably maintain their viability. This phenomenon was first termed a viable but nonculturable (VBNC) state in *Escherichia coli* and *Vibrio cholera* that were not cultivated under stress conditions [7]. For *R. solanacearum* to survive in soil or water habitats before infection of host plants, bacteria must be able to tolerate biotic and abiotic stress conditions such as nutrient competition, antibiotic production from other bacteria, UV radiation, and temperature variations. Many bacteria enter VBNC states under various environmental stresses, and VBNC induction has been reported for many non-spore-forming bacteria [8], [9], [10], [11], [12]. It has been reported that *R. solanacearum* strains also enter a VBNC state upon low temperature, starvation or copper stress [6], [13], [14], [15], [16]. VBNC induction by copper exposure or low temperature has been also demonstrated in other bacteria [17] including a plant pathogenic *Agrobacterium tumefaciens* and a legume plant symbiotic *Rhizobium leguminosarum* [18], and *Erwinia amylovora* [19].

To characterize bacterial VBNC, it is important to examine the recovery of culturability (resuscitation) of the presumed VBNC bacteria. The VBNC state of *Vibrio* strains were induced using many stress factors, and their culturability was restored when stress factors were removed [20], [21]. However, some bacteria do not return to their culturable state after elimination of the stress factors [22]. Recently, microbial autoinducers and antioxidants have been evaluated for the recovery of bacterial cell culturability [23], [24]. Attempts to resuscitate *R. solanacearum* cells in the VBNC state have been made, and amendment of sodium pyruvates was shown to revive VBNC cells [24], [35].

Gene expression in several VBNC bacteria has been investigated. The protein expression profile of *Enterococcus faecalis* in a culturable and VBNC state has been compared [26]. In *V. cholera*, mRNA expression levels in VBNC cells were monitored over time using reverse-transcription quantitative polymerase chain reaction (RT-qPCR), and the total transcriptional profile of the VBNC state was analyzed using a microarray method [27], [28]. However, changes in bacterial physiology and gene expression in the VBNC state remain poorly characterized.

The VBNC state of soil bacteria is difficult to analyze because natural soils contain various organisms and unknown compounds. Furthermore, soils from different origins are heterogeneous, which make experimental results difficult to reproduce. Therefore, a specific and uniform soil microcosm system is necessary to investigate soil microbial ecology. Ellis [28] developed the artificial soil microcosm (ASM) to model the microbial ecology of soil, which comprises the essential components of soil. In this study, we adopted this ASM to investigate the VBNC state of *R. solanacearum* strain SL341 (phylotype I, race 1). The main aim of this study was to characterize the VBNC state of *R. solanacearum* in the modified ASM (mASM) in comparison with culturable cells. We found that low temperature can induce the VBNC state of *R. solanacearum* in the soil microcosm, while catalase treatment restores the culturability of VBNC cells.

## Materials and Methods

### Bacterial strains and growth conditions

Two *Ralstonia solanacearum* strains were used to investigate the correlation between low temperature and bacterial survival in artificial soil systems. *R. solanacearum* strain SL341 was isolated from diseased tomato plants and identified as race 1, biovar 3, and phylotype I [30]. Another strain, GMI1000, was originally isolated from tomato in French Guyana, and its genome has been completely analyzed [31]. These strains were routinely cultured in casamino acid peptone glucose (CPG) broth [32] or CPG supplemented with 0.005% (w/v) 2,3,5-triphenyl tetrazolium chloride (TZC) medium at 30°C [33]. When necessary, tryptic soy agar (TSA) and nutrient agar (NA) were used to investigate bacterial culturability.

### Artificial soil microcosm and induction of a nonculturable state

Each of the components, including sand, kaolinite, bentonite, CaCO_3_ and humic acid, of the ASM were purchased from Sigma-Aldrich and used in the following quantities: 35 g, 10 g, 5 g, 0.1 g and 1 g, respectively [29]. ASM was sterilized twice by autoclaving. Modified ASM (mASM) was ASM lacking humic acid.

*R. solanacearum* SL341 and GMI1000 cells were cultured in CPG broth at 30°C for 24 h with mild shaking at 150 rpm, and were harvested by centrifugation at 16,100×*g* for 3 min. The bacterial cells were washed once with distilled sterile water, harvested again by centrifugation at 16,100×*g* for 3 min, and finally resuspended in distilled water to a density of 10^9^ CFU/mL. The mASM, which typically consisted of 50 g of the component mixture, was hydrated with 15 mL of adjusted bacterial cell suspension in 125-mL Erlenmeyer flasks and incubated at 25°C and 4°C. The bacterial cell densities were monitored over time using the dilution plating count method on CPG medium with 1 g of mASM suspended in sterile water. The experiments were conducted in triplicate.

### Total proteins and total DNA extraction from mASM

Isolation of proteins in mASM containing *R. solanacearum* SL341 was performed by modifying a metaproteome extraction and purification method [34]. Briefly, 5 g of the mASM were suspended in 10 mL protein extraction buffer (50 mM Tris-Cl (pH 8.0), 10 mM EDTA (pH 8.0), 0.5% (w/v) SDS, 1% (w/v) β-mercaptoethanol) for 30 min at room temperature, and cells in mASM were homogenized by ultrasonication (Sonic Dismembrator 500, Branson, USA). The ultrasonic wave amplitude was set at 10% of the maximum power with an irradiation pulse (10 sec on and 10 sec off) for 1 min. The suspension was mixed with 10 mL of phenol (pH 8.0) and 3 g of sucrose prior to centrifugation. The upper layer of about 10 mL of the phenol phases was transferred to a new centrifuge tube and proteins were precipitated using a fivefold volume of 0.1 M ammonium acetate in methanol at −20°C for 2 h. Protein pellets were then centrifuged at 16,100×*g* for 15 min at 4°C and washed in 80% acetone. Total proteins were dissolved in 20 mL deionized water, resolved by SDS-PAGE (14%), and visualized using Coomassie brilliant blue G-250 staining.

Total genomic DNA was isolated from culturable cells at 25°C and nonculturable cells at 4°C in mASM over time. mASM (1 g) from two temperature conditions was immediately used to isolate total DNA. DNA extraction from the soil microcosm was performed as described previously and analyzed by agarose gel electrophoresis [35]. Total DNA from mASM were quantified by Nanodrop 2000 (Thermo Fisher Scientific, MA, USA).

### Bacterial respiration analysis

Bacterial respiration was investigated using 5-cyano-2, 3-ditolyltetrazolium chloride (CTC) staining (Invitrogen, Camarillo, USA) following the manufacturer's instructions. Briefly, mASM (1 g) containing bacterial cells after incubation at different temperatures was suspended in 9 mL of sodium–phosphate buffer (pH 7.0). The culturability of bacterial cells in mASM was examined by spreading the cells on CPG agar media. When bacterial cells in mASM at 4°C were completely induced to the VBNC state by 30 days of incubation, 5 µL of a 50 mM CTC staining solution were added to 50 µL of mASM suspensions and incubated for 2 h at 37°C in the dark. The CTC-stained cell suspension was observed using an Olympus BX50 fluorescence microscope at 400× magnification. Dead bacterial cells and aseptic mASM lacking bacteria were used as negative controls. To prepare dead cells, *R. solanacearum* SL341 cells actively grown in CPG broth were harvested by centrifugation at 16,100×*g* for 10 min at room temperature. The bacterial pellet was quickly re-suspended in 70% isopropanol, incubated for 1 h, and finally harvested by centrifugation at 16,100×*g* for 10 min at room temperature [36]. The dead cells were washed and re-suspended in sodium–phosphate buffer (pH 7.0) for CTC staining.

### Resuscitation of nonculturable bacteria

To evaluate the culturability of bacterial cells in mASM, mASM (1 g) containing nonculturable cells was suspended in 10 mL of distilled water, and 100 µL of the suspension were spread on different complex media for *R. solanacearum*, such as CPG, TZC and TSA agar media. To confirm the absence of culturable cells in the mASM with SL341 at 4°C (which causes re-growth of a small number of culturable cells present in the mASM), 10 mL of soil supernatant containing the bacterial cells were recovered from soil particles by pipetting. The bacterial cells in the suspension were subsequently harvested by centrifugation at 16,100×*g* for 5 min. The pellet was resuspended in 1 mL of sterile water and the suspension was spread on several CPG, TZC, and TSA agar plates to confirm that no *R. solanacearum* colonies formed.

Recovery of nonculturable bacterial cells after a temperature increase was performed as follows; 1 g of mASM containing nonculturable bacteria cells was suspended in 10 mL of sterile water and incubated at 30°C for 3 days. The sample suspension exposed to 30°C was investigated using the dilution plating count method described above. To examine their effects on nonculturable cells in mASM, we supplemented the suspension with the following compounds: thiamine hydrochloride, catalase, sodium pyruvate, CPG broth, V8 juice and acyl-homoserine lactone (AHL). Briefly, sodium pyruvate (0.2% (w/v)), thiamine hydrochloride (1.5 mg/mL) and 100 µM AHLs (3-oxo-C_8_-homoserine lactone (HSL), C_8_-HSL, and C_6_-HSL) were added directly to the sterile water. mASM (1 g) was resuspended in 10 mL of sterile water containing the compounds mentioned above. The negative control was mASM suspended in sterile water lacking chemical supplements. The soil microcosm suspensions were incubated at 30°C for 3 days, and bacteria were enumerated using the dilution plating count method on several culture media.

### Plant growth and bacterial virulence assays

Tomato cultivar Moneymaker was used for bacterial virulence assays. The surface sterilized tomato seeds were germinated and tomato plants were grown in pots containing commercial horticulture nursery-media soil (Punong Co., Ltd, Korea) and maintained in the growth chamber for 3 weeks at 28°C. The virulence of the resuscitated cells and culturable SL341 strain were investigated in 3-week-old tomato plants ‘Moneymaker’ using soil soaking inoculation [32]. Bacterial inoculums were prepared by growing bacterial cells in CPG broth at 30°C for two days and resuspending bacterial cells in sterile water. The inoculum were adjusted the O.D.600 of 0.5 (4∼5×10^8^ CFU/mL) with distilled water. For soil soaking method, each tomato was poured bacterial suspension with the final concentration ∼10^7^ CFU/g soil. A total of randomly selected 10 different resuscitated bacterial strains used for bacterial virulence assay with their wild type strain SL341. All inoculations included fifteen plants for each strain and the non-inoculated controls with three replications. All inoculated plants were incubated in a growth chamber at 28°C in light for 14 h and in the dark for 10 h. Plants were monitored for disease progress over time after inoculation, and bacterial wilt was rated using the following scale: 0, no wilting; 1, 1–25% leaves wilted; 2, 25–50 leaves wilted; 3, 51–75% leaves wilted; 4, 76–100% leaves wilted.

### Effect of catalase on bacterial resuscitation

The resuscitation of nonculturable cells by catalase was performed as follows. mASM (1 g) with nonculturable cells was inoculated into 10 mL of sterile water or yeast peptone (YP) medium (yeast extract, 0.25% (w / v); peptone, 0.5% (w / v)), and the suspension was divided into two tubes. One of the tubes was supplemented with catalase to a final concentration of 1,000 U/mL and incubated at 30°C for 3 days; bacteria were then enumerated using the plate count method. Catalase from bovine liver was purchased from Sigma Corp., suspended in double-distilled water, and sterilized by passing through 0.2-µm pore size membrane filters.

Bacterial resuscitation was investigated using catalase treatment in mASM containing *R. solanacearum* SL341 at both 25°C and 4°C. Sterile water with or without autoclaved catalase (inactivated catalase) were used as negative controls. mASM (1 g) with SL341 incubated at 25°C and 4°C was suspended in distilled sterile water. The suspension was divided into three tubes; two were supplemented with catalase or autoclaved catalase and incubated for 3 days at 30°C. Bacteria were then enumerated using the plate count method on CPG medium following incubation for 2 days. The experiments included three replicates.

### RNA extraction from the soil microcosm and reverse-transcription quantitative PCR (RT-qPCR)

The RNA expression levels of *R. solanacearum* SL341 in mASM were determined by RT-qPCR. Bacterial total RNA was isolated using the Hybrid-R RNA extraction kit (GeneAll Bio Inc., Seoul, Korea) from 1 g of mASM at various stages. The RNA was eluted in 30 µL of RNase-free water, and 5 µL were directly used as template for cDNA synthesis by the Prime Script RT Master Mix kit (TaKaRa Biotech. Co., Japan), following the manufacturer's instructions. Potential contamination of DNA in the RNA was excluded by DNaseI treatment prior to cDNA synthesis. Quantitative PCR using the cDNA was conducted using a CFX384 Real Time System (Bio-Rad, Hercules, CA, USA). The qPCR reaction components contained SYBR Premix Ex Taq mix (TaKaRa Biotech. Co., Japan), 1 µL of diluted cDNA template, 10 µM both forward and reverse primers (Table S1), and RNase free water. Thermal cycling included two reaction steps; an initial preheat for 3 min at 95°C, followed by 40 cycles at 95°C for 5 sec, 60°C for 10 sec (50°C for 10 sec for the *V*3 primers), and 72°C for 35 sec. The qPCR data were displayed using the CFX manager software version 1.6. The oligonucleotide primers were designed based on the *R. solanacearum* GMI1000 sequence (Table S1).

Standard curves between the cDNA concentration and C_(t)_ value of qPCR for individual genes were obtained using various DNA concentrations of target DNA for qPCR. First, target DNA was obtained by PCR using the indicated primers (Table S1), and the concentration of the purified PCR product was measured using a NanoDrop 2000 spectrophotometer (Thermo Scientific, Wilmington, USA). The template DNA was diluted serially 10-fold to various concentrations, and the corresponding C_(t)_ value was obtained by qPCR. Finally, standard curves were generated by plotting DNA concentrations and their corresponding C_(t)_ values for individual genes.

RT-qPCR results of individual genes were evaluated using the iCycler iQ Real-Time PCR Detection System. The C_(t)_ values of qPCR products of each gene were used to determine the target cDNA concentration based on the standard curves. The molar ratio of cDNA copy numbers of each gene was determined using the following equation:

The molar ratio of cDNA copies  =  the amount of cDNA (ng)/the molecular weight of expected PCR product of dsDNA (ng)

The amount of cDNA was determined based on the cDNA concentration after multiplying by the fold-dilution of each gene. The molecular weight of the expected PCR product was calculated using the Sequence Manipulation Suite software (http://www.bioinformatics.org/sms2/index.html). The cDNA copies for each gene of *R. solanacearum* in mASM were compared to the number of cDNA copies of the 0 day bacterial incubation in mASM at 25°C. The RT-qPCR experiments included three replicates.

## Results

### Bacterial viability in mASM at two temperatures

**Figure 1:**
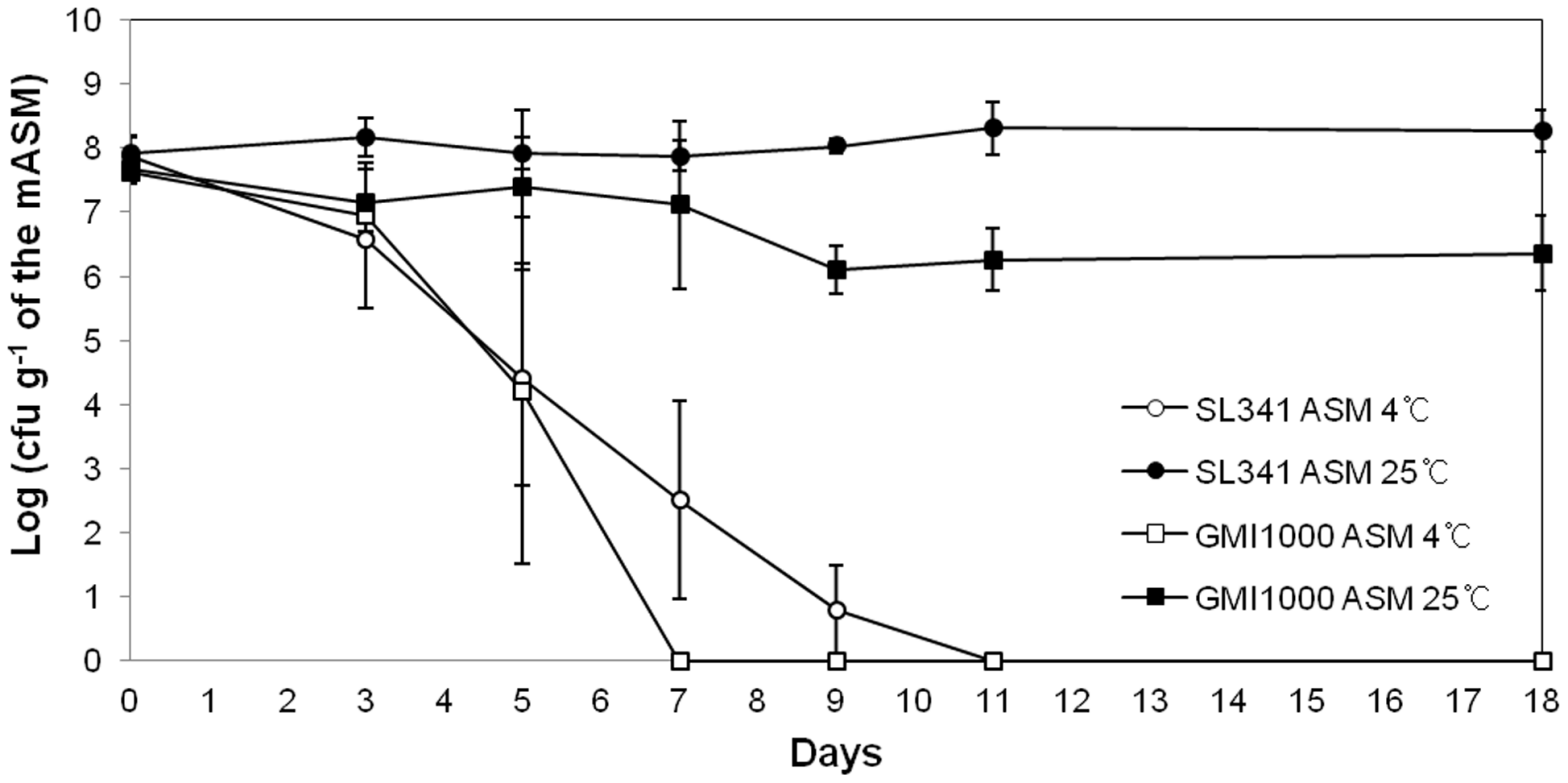
Culturability of *R. solanacearum* in a modified artificial soil microcosm over time. *R. solanacearum* SL341 (circle) and GMI1000 (square) in the microcosm at 25°C (closed circle and closed square) or 4°C (open circle and open square). Vertical bars represent standard deviations of three replicates.

We adopted the ASM system to investigate *R. solanacearum* survival in soil. ASM was first developed by Ellis [29] to contain the majority of essential soil components. However, the presence of humic acid, which is a nutrient component of ASM, from different manufacturers (or even the same company) resulted in inconsistent survival rates of *R. solanacearum* SL341 and GMI1000. Therefore, we modified the ASM by excluding humic acid, and referred to the modified ASM as mASM. Interestingly, culturable cells of strain SL341 were stably maintained at 25°C in mASM for 13 days. In the same system, culturable *R. solanacearum* GMI1000 strain also maintained its population at 25°C for 13 days, albeit with a slightly reduced cell density (Fig. 1). Since low temperature stress induces the nonculturable state of *R. solanacearum* in water [14], we inoculated *R. solanacearum* SL341 and GMI1000 in mASM, incubated at 4°C, and enumerated culturable bacterial cells. The culturability of SL341 and the GMI1000 decreased over time at 4°C. Finally, no culturable SL341 or GMI1000 cells were detected on nutrient agar, CPG, or minimal medium after 11 days and 7 days of incubation in mASM (Fig. 1). This suggested that mASM could be used to investigate the ecology of *R. solanacearum* strains and its survival at different temperatures.

### Viability of nonculturable bacteria

**Figure 2:**
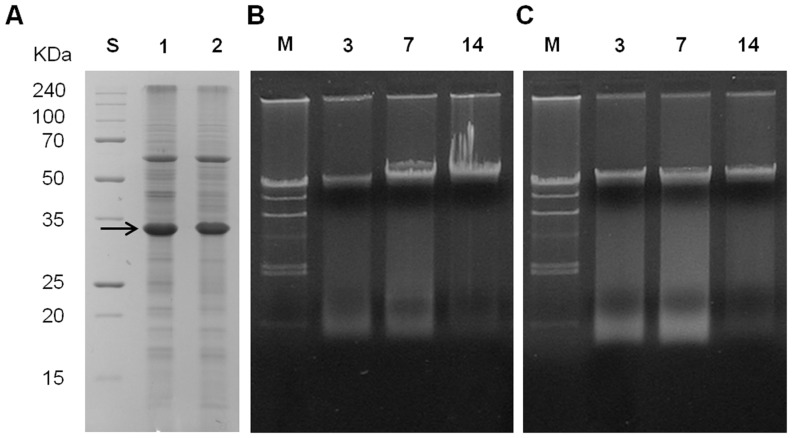
Total protein profile and total DNA isolated from mASM. (A) Total proteins purified from culturable and nonculturable *R. solanacearum* in mASM examined by SDS-PAGE; S, protein size standard; 1, culturable *R. solanacearum*; 2, nonculturable *R. solanacearum*. (B) and (C) Total DNA isolated from mASM at 25°C and 4°C, respectively. M, DNA size marker λ-DNA digested with *Hind*III; 1, 3-day incubation of bacteria in mASM; 2, 7-day incubation of bacteria in mASM; 3, 14-day incubation of bacteria in mASM. The arrow indicates the Omp protein which was constitutively produced in both bacterial cells.

**Figure 3:**
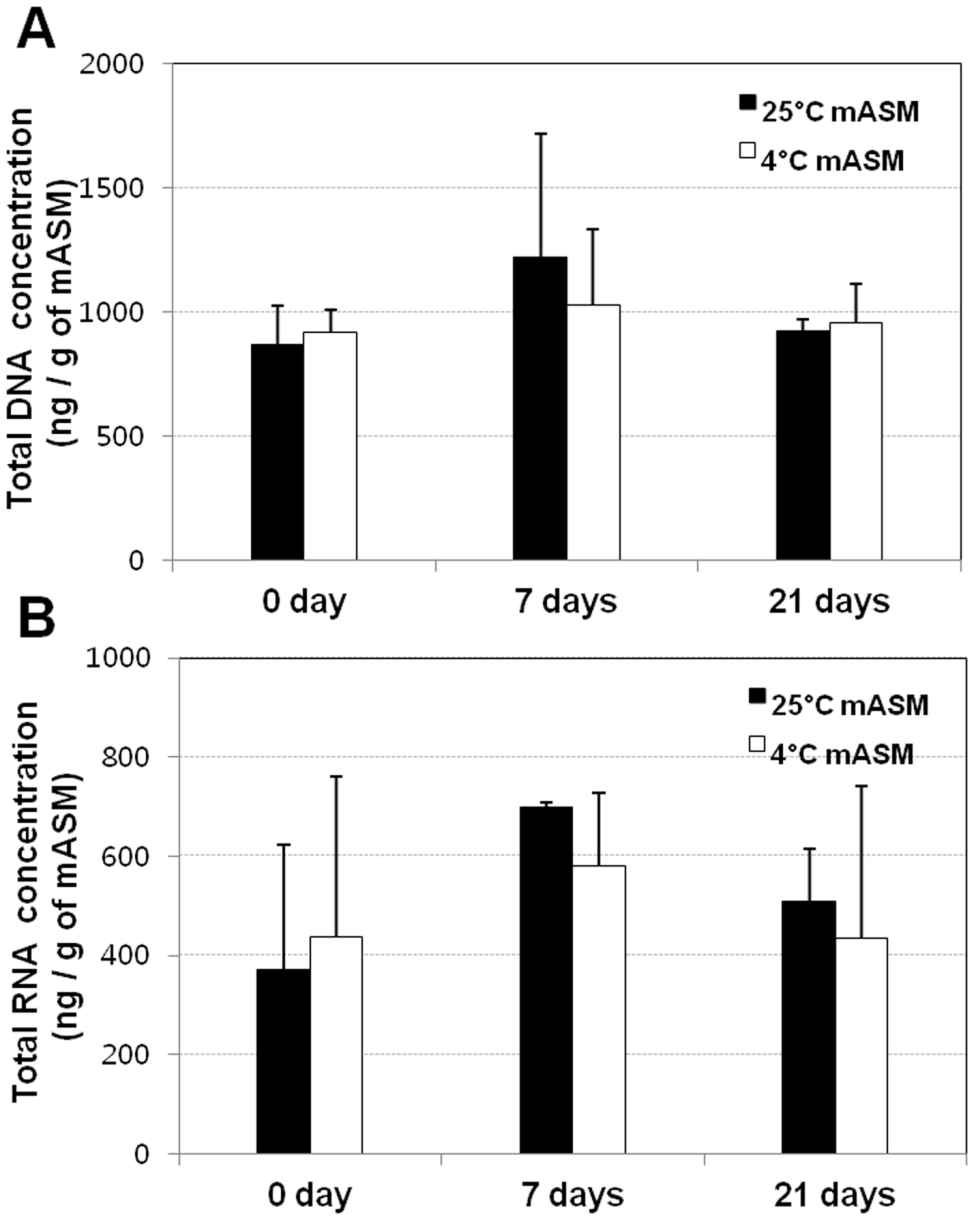
Quantification of total DNA and RNA in mASM at 25°C and 4°C. (A) Total DNA purified from mASM, (B) total RNA isolated from mASM at 25°C and 4°C at 0, 7 and 21 days after incubation.

**Figure 4:**
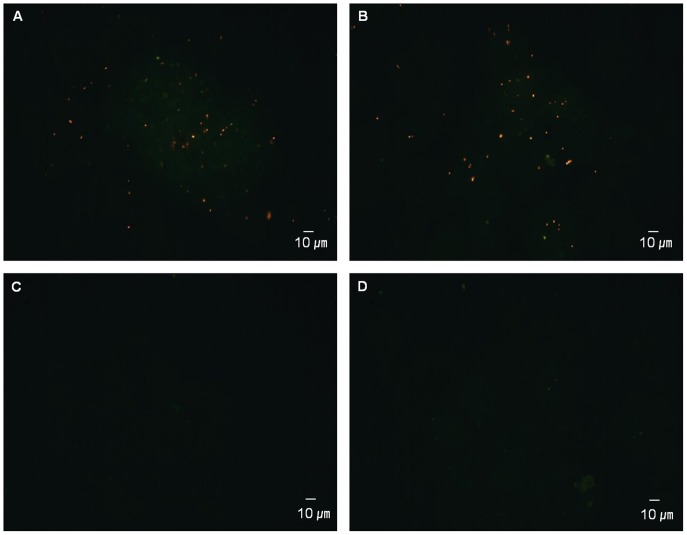
Fluorescence micrograph of CTC-stained bacteria in mASM (magnification, ×100). (A) Culturable cell control maintained in mASM for 30 days at 25°C, (B) VBNC cells maintained in mASM for 30 days at 4°C, (C) dead cells treated with 70% isopropanol for 2 h, and (D) mASM control without bacterial cells.

Because low temperature (4°C) suppresses the culturability of *R. solanacearum* strains, cells at such low temperatures may be dead. To determine whether the bacteria are alive or dead at low temperatures in mASM, we compared total protein patterns and total DNA of *R. solanacearum* SL341 in mASM incubated at two temperatures. SDS-PAGE of total protein showed that the majority of proteins remained intact in *R. solanacearum* SL341 incubated for 14 days in mASM at both 25°C and 4°C, although the levels of some proteins were decreased slightly at 4°C (Fig. 2A). The content and size of total DNA of *R. solanacearum* SL341 in mASM at 25°C or 4°C were not remarkably different after incubation for 3, 7 and 14 days (Fig. 2B and C). When total DNA and RNA from the microcosm were quantified, any remarkable difference was not apparent between culturable state and nonculturable state (Fig. 3). Bacterial respiration is an important indicator of viability. We adopted the CTC staining method to investigate bacterial respiration in mASM. CTC staining has been used widely to examine bacterial respiration in various microbial communities [37], [38] CTC responds to the bacterial electron-transport chain and becomes reduced to form formazan precipitates (CTF), which generates red fluorescence. Our analysis revealed the presence of respiring bacterial cells in mASM suspension after incubation with CTC solution for 2 h. Respiration was also apparent in bacterial cells in a culturable (25°C) and VBNC state (4°C) in mASM for 30 days (Fig. 4). The number of respiring bacterial cells (red fluorescence) in the VBNC state (Fig. 4B) was similar to that of respiring culturable cells (Fig. 4A). We confirmed complete loss of culturability of VBNC cells maintained in mASM for 30 days at 4°C, while the number of culturable bacterial cells in mASM for 30 days at 25°C was 2×10^8^ CFU/g of mASM. However, we could not detect respiration of dead cells in suspension or aseptic mASM lacking bacterial cells (Fig. 4C and D).

### Catalase treatment resuscitates nonculturable bacteria

**Figure 5:**
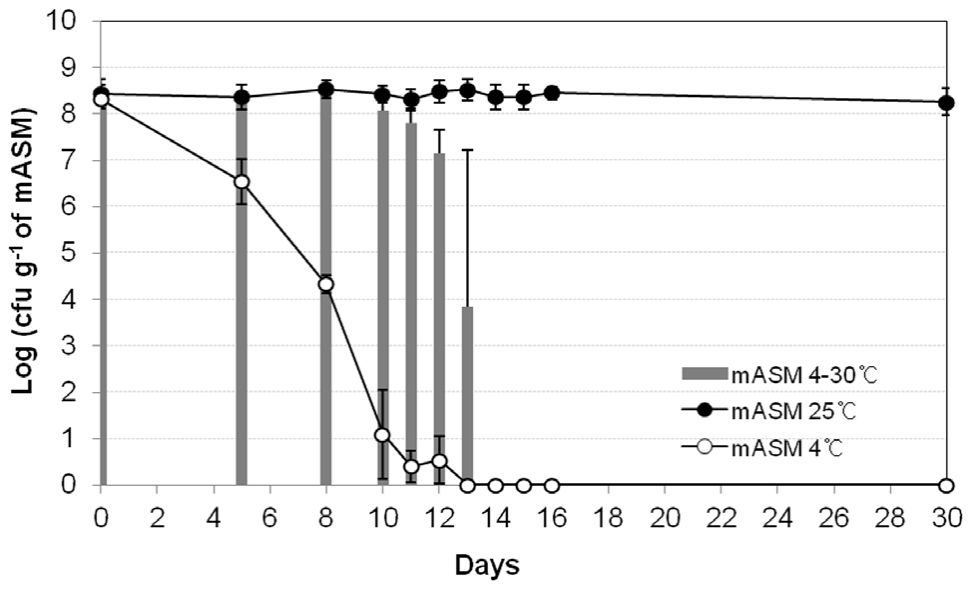
Resuscitation of VBNC *R. solanacearum* SL341 cells by an increase in temperature. VBNC cells were incubated at 30°C for 3 days and then culturable cells were enumerated based on the formation of visible colonies on CPG medium (thick gray bar). Culturable cells (closed square) and nonculturable cells (open square). Vertical bars represent standard deviations of three replicates.

Recovery of viable bacterial cells (resuscitation) is used to determine the viability of *R. solanacearum* SL341 [39] at low temperatures in mASM. Therefore, we used several methods to resuscitate nonculturable SL341 cells in mASM, including supplementation and incubation for up to 3 days in the presence of CPG broth, V8 juice, tomato extract, thiamine hydrochloride, autoinducers such as AHLs, and pyruvates. None of the supplements promoted resuscitation of VBNC cells in mASM. An increase in temperature to 30°C in mASM did not result in resuscitation of nonculturable *R. solanacearum* SL341 cells at 14 days after incubation in mASM at 4°C (Fig. 5). However, an increase in the temperature of mASM containing a small number of culturable cells resulted in bacterial re-growth (see day 13, Fig. 5).

**Figure 6:**
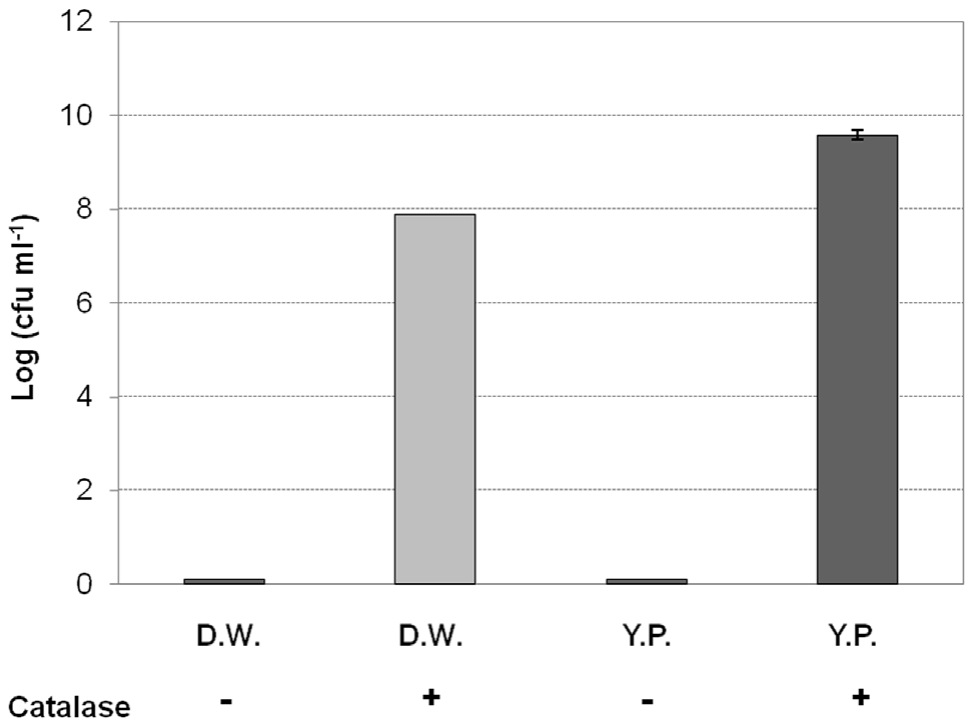
Resuscitation of VBNC cells with or without catalase. VBNC cells in distilled water (D.W.) and nutrient-rich medium (YP) were treated with catalase and incubated at 30°C for 3 days. Culturable cells were then enumerated based on the formation of visible colonies on CPG medium. Vertical bars represent standard deviations of three replicates.

**Figure 7:**
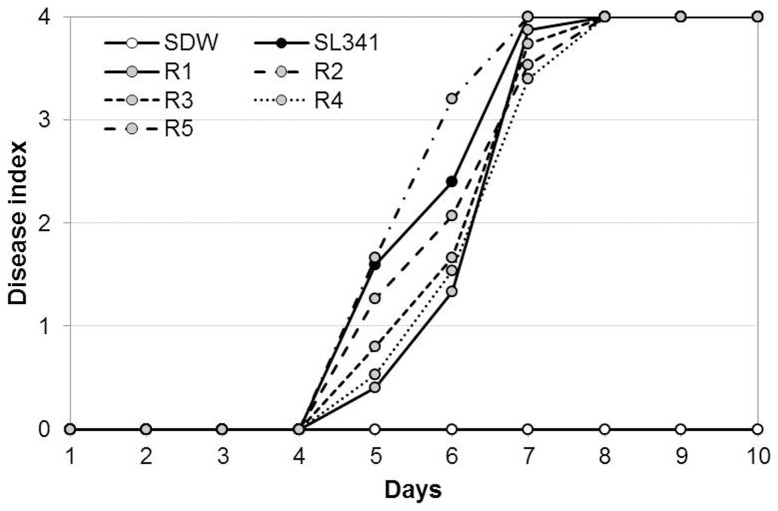
Disease progress by resuscitated cells on tomato plants. Culturable *R. solanacearum* SL341 (closed circle), resuscitated strains (gray circles, R1∼5) and sterile water as control (opened circle) were inoculated using soil soaking. Vertical bars represent standard errors of three replication (n = 15 each).

Interestingly, catalase treatment resuscitated nonculturable *R. solanacearum* SL341 incubated in mASM at 4°C. Briefly, mASM containing nonculturable bacteria was suspended in YP broth or distilled water supplemented with 1,000 U/mL catalase. Following incubation at 30°C for 1 to 3 days, nonculturable SL341 formed bacterial colonies on TZC or TSA medium, indicative of resuscitation (Fig. 6). However, bacterial cells from the same mASM suspension lacking catalase did not form colonies (Fig. 6). The recovered cell density was similar to the initial inoculum in the presence of catalase, while the cell density in YP broth with catalase increased compared to the inoculum, (Fig. 6). The catalase treatment also showed the resuscitation of GMI1000 strain (data not shown). Moreover, all of the resuscitated cells maintained full virulence by soil soaking inoculation compared with culturable SL341 wild type strain to 3-week-old tomato plants (cv. Moneymaker), which is a tomato cultivar susceptible to bacterial wilt (Fig. 7).

### Three phases of the nonculturable state of *R. solanacearum*

**Figure 8:**
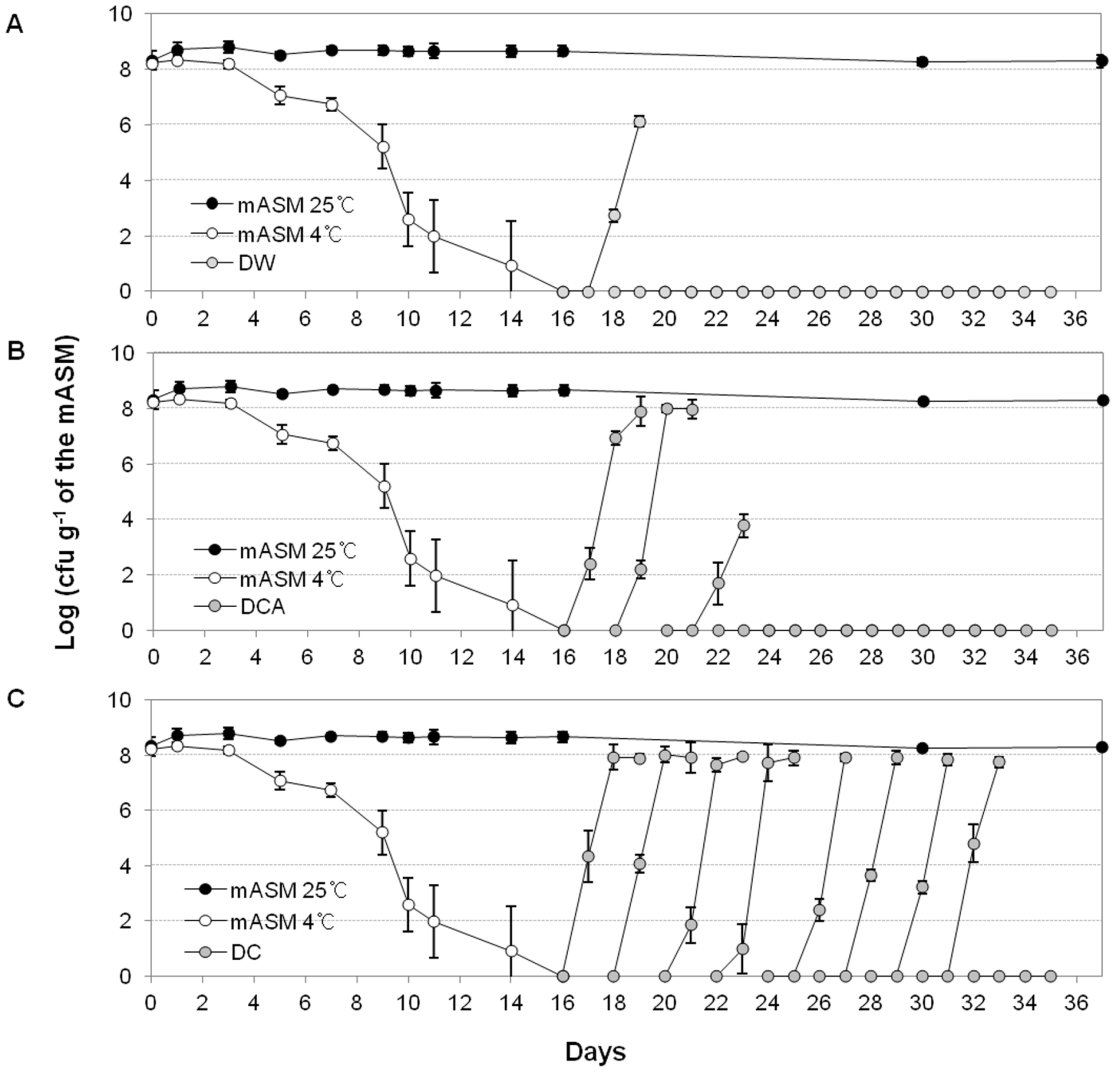
Resuscitation of VBNC *R. solanacearum* SL341 cells. Culturable cells were enumerated based on the formation of visible colonies on CPG medium. The filled circles and open circles represent the number of culturable bacterial cell from mASM at 25°C and 4°C over time, respectively. The gray circles in panel A, B, and C represent the number of culturable bacterial cells incubated with sterile water without catalase (DW), with autoclaved catalase (DCA), and with native catalase (DC) for 36 days, respectively. Vertical bars represent standard deviations of three replicates.

Resuscitation of SL341 in mASM by catalase over time was investigated. Nonculturable bacteria SL341 in mASM were not resuscitated by incubation in sterile water lacking catalase or with autoclaved catalase, although some resuscitated cells were present until 4 days after incubation in mASM at 4°C (Fig. 8A and B). It is likely that the resuscitated bacterial cells 4 days after VBNC induction were not in a true nonculturable state. Bacterial cells at 5 days after induction of the nonculturable state were not resuscitated by water alone or water containing autoclaved catalase, but were resuscitated 16 days after the addition of catalase (Fig. 8C). Therefore, the true nonculturable state of SL341 in mASM occurs ∼12 days after transition to the nonculturable state. Moreover, nonculturable cells cannot be recovered to the culturable state by incubation with catalase for 12 days (after 30 days at 4°C in mASM) (Fig. 8C).

Therefore, the VBNC state can be divided into three phases: Phase I, pre-nonculturable stage (which still contains small number of culturable bacteria); Phase II, nonculturable stage (resuscitated by catalase); and Phase III, nonculturable stage (not resuscitated by catalase).

### Gene expression by culturable and nonculturable bacteria

**Figure 9:**
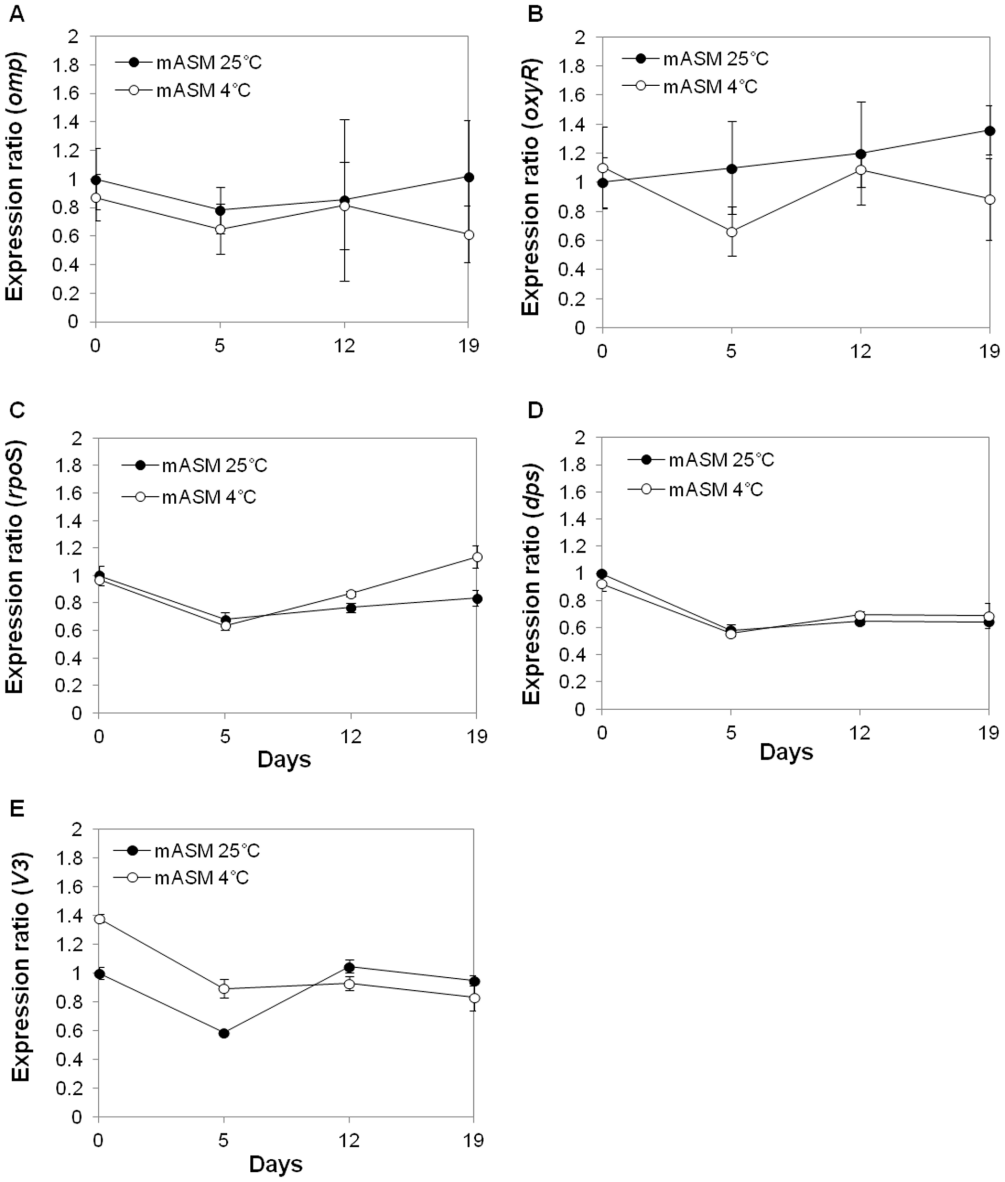
Gene expression in *R. solanacearum* SL341 in mASM at 25°C and 4°C by RT-qPCR. Total RNA was isolated from mASM at 0, 5, 12 and 19 days after incubation in mASM. Expression levels of *omp* (A), *oxyR* (B), *rpoS* (C), *dps* (D), and the *V3* region of the 16S rRNA gene (E), were estimated in comparison to those at day 0 at 25°C in mASM. Vertical bars represent standard deviations of three replicates.

Gene expression by culturable and nonculturable bacteria at 25°C and 4°C in mASM was investigated by RT-qPCR. The stages for comparison included the initial SL341 inoculum, pre-nonculturable stage, and nonculturable stage, during which SL341 can be resuscitated by catalase. The *omp* gene was used as a positive control because it was strongly expressed as determined by detection of the product using SDS-PAGE (Fig. 2A, the arrow) and protein identification (data not shown). mRNA expression of *omp, oxyR*, *rpo*S, *dps*, and the *V*3 region of 16S rRNA gene differed slightly between the two temperatures (Fig. 9). However, no significant differences in gene expression at the transcriptional level between bacterial cells at 25°C and 4°C in mASM were observed.

## Discussion

The nonculturable or VBNC state of *R. solanacearum* has been reported in watercourses, liquid microcosms and soil, and can be induced by low temperature, starvation or copper stress [5], [13], [14], [15], [16]. However, the VBNC state is not well understood. Here, we used an artificial soil microcosm (ASM) system [29] to investigate the nonculturable state of *R. solanacearum*. Previously, sterilized environmental soil has been used to investigate the microbial ecology of soil [40], [41] because it mimics natural soil in terms of its physico-chemical composition. Nonetheless, sterilized natural soil lacks reproducibility due to the variable characteristics of soils from different origins. Ellis [29] first established ASM by mixing commercial components of soils and showed that *Pseudomonas* strains stably maintained their cell density. In this study, ASM was used to study the nonculturable state of *R. solanacearum* strains in soils.

The viability of *R. solanacearum* strains SL341 and GMI1000 in ASM at 25°C was maintained for over 1 month. However, the culturable cell population of SL341 and GMI1000 varied due to the presence of humic acid, which is included in ASM as a nutrient (data not shown). The variability of culturable cell density in ASM may be due to use of humic acid of different origins because it is an organic compound [42]. Interestingly, both *R. solanacearum* strains GMI1000 and SL341 stably and reproducibly maintained their cell density in mASM (ASM without humic acid) under unstressed conditions. *R. solanacearum* strains can survive for extended periods of time in soil lacking nutrients [6]. The reproducible survival of the *R. solanacearum* strains suggests that *R. solanacearum* ecology can be investigated using mASM, which mimics the bacterial ecology of soil.

When mASM containing *R. solanacearum* SL341 and GMI1000 was incubated at 25°C or 4°C, the culturability of both strains was similar. While the proportion of culturable bacteria was maintained at 25°C, that at 4°C decreased over time (Fig. 1). It has been reported that while *R. solanacearum* population decreased during the winter season in soil or water microcosm, the culturable cell density increased with increasing temperature [13], [14]. This phenomenon is due to the presence of VBNC *R. solanacearum* cells. Therefore, the loss of culturability of *R. solanacearum* at 4°C could result from bacteria entering the VBNC state. Although the nonculturable bacterial cells were unable to form colonies on regular culture media, maintaining total DNA and proteins in these cells suggested that they retain fundamental metabolism. However, the intact DNA and proteins in mASM incubated at 4°C cannot be taken as direct evidence of *R. solanacearum* cell viability.

The resuscitation of VBNC cells in mASM indicates their viability. *Vibrio* strains in the VBNC state induced by low temperature were recovered by removing stress factors, such as increasing the temperature [20], which indicated their viability. We also attempted to resuscitate VBNC cells by increasing the temperature. Our results revealed that a temperature increase (before complete loss of culturability) recovered bacterial culturability (Fig. 5). However, VBNC cells in mASM did not recover culturability by a simple temperature increase. This result suggested that the culturability of nonculturable cells in mASM could not be restored by increasing the temperature. The addition of vitamin B_1_, sodium pyruvate, nutrients (CPG, V8 juice) and AHLs has been used to recover bacterial culturability since these are known to resuscitate VBNC cells of other bacterial taxa [21], [23], [24], [43], [44]. However, none of the additives resuscitated SL341 cells in mASM.

Recently, it was reported that the oxidative stress response in bacteria is associated with cold stress [45], and VBNC cells of *Vibrio*, *Ralstonia* and *Escherichia* induced by low temperature were successfully resuscitated by treatment with antioxidant agents [21], [24], [25], [46]. Our results also showed that culturability of VBNC cells of *R. solanacearum* SL341 was recovered in the presence of catalase (Fig. 6), which indicates that oxidative stress induced internally or externally may be important for induction of the VBNC state of *R. solanacearum*. However, sodium pyruvate, which protects against H_2_O_2_, did not resuscitate nonculturable bacterial cells, despite the fact that supplementation with sodium pyruvate in mASM or TZC medium delayed induction of the VBNC state in both GMI1000 and SL341 strains (data not shown). Therefore, it is likely that sodium pyruvate has a minor effect on the resuscitation of VBNC cells. The resuscitation of VBNC SL341 and GMI1000 cells by catalase treatment was reproducible. To exclude the possibility that catalase itself serves as a nutrient to support the re-growth of culturable SL341 cells, autoclaved catalase was used as a negative control (Fig. 8). The resuscitation of VBNC SL341 cells by catalase (but not by autoclaved catalase) suggested hydrogen peroxide accumulation in cells exposed to low temperature stress and suppression of colony formation on regular culture medium. It remains unclear whether the resuscitation of VBNC cells corresponds to re-growth or resuscitation [47], [48]. The number of bacterial cells resuscitated by distilled water containing catalase was equal to the initial inoculum. Failure of resuscitation in the presence of autoclaved catalase indicated that catalase itself does not support the re-growth of culturable cells. Since mASM contains no nutrient that would support the growth of culturable cells, resuscitation is the result of recovery of the culturability of VBNC *R. solanacearum* SL341 cells. The recovered SL341 cells showed similar phenotypes to the original strain, including virulence towards tomato plants. Since GMI1000 VBNC cells showed the similar resuscitation by catalase treatment, our mASM microcosm system could be generally used for VBNC state and its resuscitation of phylotype I strains.

H_2_O_2_ can permeate through bacterial cell membranes, although the permeability is not unlimited [49]. H_2_O_2_ produced in SL341 cells in response to low temperature stress might have accumulated to a critical concentration extra- or intracellularly, which suppressed colony formation on culture media. In fact, copper-treated *R. solanacearum* SL341 lost culturability and accumulated H_2_O_2_ compared to cells in the culturable state [16]. Therefore, an external supply of catalase, which removes H_2_O_2_ in mASM and cells, may reduce the intracellular H_2_O_2_ concentration, thus facilitating colony formation on regular culture media. The resuscitation of cells exposed to low temperature stress by catalase has been reported for other bacterial taxa [20], [21]. Time-course experiments of resuscitation of *R. solanacearum* SL341 VBNC cells suggested the following three phases of SL341 cell culturability at 4°C in mASM: Phase I, pre-nonculturable stage; Phase II, nonculturable stage can be resuscitated by catalase; Phase III, nonculturable stage but cannot be resuscitated by catalase. The failed resuscitation of VBNC cells by catalase in phase III may be due to entry to a death stage. Otherwise, other stress factors induced by the low temperature (other than H_2_O_2_) might also have affected the cells. It remains unclear whether phase III represents a bacterial death stage. SL341 cells could be injured by H_2_O_2_ in this phase [39], and thus may not be resuscitated by its removal. Interestingly, the nonculturable bacterial cells in phase III were respiration-positive based on CTC staining (Fig. 4).

To assess gene expression in VBNC cells, the *V*3 region of 16S rRNA and *omp* genes were used as a positive control for expression in both culturable and nonculturable cells. *Omp* was selected because an Omp protein was produced constitutively in both culturable and VBNC cells (see the major protein at ca. 32 kDa in Fig. 2A). The *rpoS*, *oxyR* and *dps* genes were selected because they are involved in regulating the expression of genes associated with bacterial survival and the stress response [50], [51], [52]. The *rpoS* and *oxyR* genes are involved in the production of antioxidant enzymes [51], [53], and the *dps* gene encodes a starvation- or oxidative-stress-induced DNA-binding protein in *Escherichia coli* [54]. Although there is no difference in the expression of those genes between culturable cells and VBNC cells, their constitutive expression of those genes in VBNC SL341 cells may suggest continued transcription of essential transcripts and stable maintenance of their transcript level (Fig. 9).

In conclusion, we used the mASM system to demonstrate that low temperature induced the VBNC state of *R. solanacearum*. Moreover, VBNC *R. solanacearum* SL341 cells maintains their respiration, gene expression and protein production, and could be resuscitated by catalase treatment. VBNC is considered a survival strategy for pathogenic bacteria, such as *Ralstonia*, in the soil. Therefore, elimination of VBNC *R. solanacearum* cells could be a novel approach to controlling or preventing plant infection by harmful microorganisms.

## Supporting Information

Table S1 **Oligonucleotides used for RT-qPCR.** *Annealing temperature for PCR amplification.
(DOCX)
