# Supplementary material for: Induction of the Viable but Nonculturable State of Ralstonia solanacearum by Low Temperature in the Soil Microcosm and Its Resuscitation by Catalase
Source: PLoS One. 2014 Oct 8;9(10):e109792. doi: 10.1371/journal.pone.0109792 (PMC4190316; doi:10.1371/journal.pone.0109792)
Supplement: Table S1 — Oligonucleotides used for RT-qPCR. *Annealing temperature for PCR amplification. (DOCX) [file pone.0109792.s001.docx]

**Supporting information**

**Table S1.** Oligonucleotides used for RT-qPCR.

| Gene | Forward (5’- 3’) | *Ta (°C) | Amplicon size  (bp) |
| --- | --- | --- | --- |
|  | Reverse (5’- 3’) |  |  |
| *omp* | F: GCGCTGGCAACCTGCGTTCG | 60 | 166 |
|  | R: GTGCGCTTGGACAGCGCGTA |  |  |
| *oxyR* | F:ACCAGCCGCGCGGTGAAGTTT | 60 | 101 |
|  | R:ACGATCGGGCCGTACCTGCT |  |  |
| *rpoS* | F:AGGTGATGATCGAGCGCA | 60 | 270 |
|  | R:TGGCGCGCAGCACCTGGT |  |  |
| *dps* | F:GGACCGCCGTGGATTCGGTGG | 60 | 189 |
|  | R:CCGGGAACAGGCTGCGTGCG |  |  |
| *V3* of 16S rRNA | F:ACTCCTACGGRAGGCAGCAG | 50 | 197 |
|  | R:ATTACCGCGGCTGCTGG |  |  |

*Annealing temperature for PCR amplification.
